# parkrun and the promotion of physical activity: insights for primary care clinicians from an online survey

**DOI:** 10.3399/BJGP.2022.0001

**Published:** 2022-08-23

**Authors:** Steve Haake, Helen Quirk, Alice Bullas

**Affiliations:** The Advanced Wellbeing Research Centre, Sheffield Hallam University, Sheffield.; National Institute for Health Research School for Public Health Research launching fellow in public health, School of Health and Related Research, University of Sheffield, Sheffield.; The Advanced Wellbeing Research Centre, Sheffield Hallam University, Sheffield.

**Keywords:** exercise, health promotion, long-term health conditions, parkrun, running, social prescribing, walking

## Abstract

**Background:**

To support efforts to increase social prescribing and reduce levels of physical inactivity, parkrun UK and the Royal College of General Practitioners together developed the parkrun practice initiative to link general practices to local parkruns (free, weekly, timed, physical activity events). General practice staff are encouraged to take part in parkrun events themselves and to encourage patients to participate.

**Aim:**

To provide insights for primary care clinicians about parkrun participants (parkrunners), especially those with characteristics of patients who might be signposted to physical activity.

**Design and setting:**

Secondary analysis of an online survey of parkrunners in the UK.

**Method:**

Responders were ranked into 13 categories using mean parkrun finish time, allowing the following definitions: front runners; median runners; slower runners; runners/walkers; and walkers. Measures included demographics, health conditions, motives for first participating, and perceived impact on health and wellbeing.

**Results:**

The survey included 45 662 parkrunners. More than 9% of all participants and 45% of walkers were found to have at least one long-term health condition, including arthritis, obesity, depression, hypertension, chronic pain, anxiety, type 2 diabetes, and asthma. Walkers were less likely to be motivated by fitness or competition, and were more likely to be motivated by physical health. Despite these differences, perceived improvements to wellbeing were broadly similar for all parkrunners, regardless of their finishing time.

**Conclusion:**

Parkrunners are a diverse population in terms of their physical health. Information provided by this study could be combined with other research on the barriers to participation and successful brief interventions to help address the key issues of primary care clinicians’ knowledge and confidence about social prescribing to increase patients’ physical activity levels.

## INTRODUCTION

Guidance from the UK’s Chief Medical Officers (CMO) recommends at least 150 min of moderate or 75 min of vigorous physical activity per week to optimise health outcomes.^1^ Worldwide, one in four adults and three in four adolescents do not meet these activity guidelines.^2^ Primary care clinicians are advised to carry out social prescribing rather than clinical interventions to increase patients’ physical activity,^3^ and one-quarter of patients say that they would be more active if advised by a nurse or GP.^4^ The conditions that GPs would refer physical activity for include: type 2 diabetes, depression, anxiety, hypertension, arthritis, obesity, and being overweight.^5^

Previous research showed that up to 70–80% of GPs do not speak to their patients about physical activity,^6^ while 80% are unfamiliar with the CMO physical activity guidance.^5^ As part of its *Global Action Plan on Physical Activity 2018–2030*,^2^ the World Health Organization (WHO) identified that mass participation initiatives in public spaces that engage whole communities could provide *‘enjoyable, affordable and culturally appropriate experiences of physical activity’*. Parkrun *,* a charity that puts on free, weekly, timed 5 km events across 23 countries was suggested by the WHO as a good example of such an initiative.^3^^,^^7^^,^^8^

Given the need to increase social prescribing and reduce physical inactivity (as set out in *The NHS Long Term Plan* ^9^), the parkrun practice initiative was created jointly by parkrun UK and the Royal College of General Practitioners, to support these efforts by linking primary care with a physical activity opportunity (parkrun).^10^^–^^12^ However, there is a lack of understanding among both patients and clinicians about what parkrun is and the appropriateness for some patients of participating in it.

This study is a secondary analysis of a health and wellbeing survey of parkrunners carried out in 2018.^13^ The aim of the study was to provide insights for primary care clinicians about the benefits of social prescribing for patients by outlining the broad range of people that take part in parkrun as walkers or runners, and describing whether they have long-term health conditions, what motivates them to first participate, and the impact of their participation.

## METHOD

The survey employed a mix of validated measures used in health and wellbeing research, using questions created by a team of academics and health practitioners.^13^ It was sent via parkrun using Qualtrics to all 2 318 135 registered parkrunners aged ≥16 years between 29 October and 3 December 2018.

**Table:** How this fits in

To support efforts to increase social prescribing and signposting to physical activity opportunities, the Royal College of General Practitioners and parkrun UK developed the parkrun practice initiative, which, so far, has seen more than 1500 general practices link with their local parkrun events (free, weekly, timed 5 km events). Not all GPs and primary care clinicians are confident in prescribing physical activity and this study aimed to provide useful insights from a large survey of parkrunners. A total of 9.3% of responders had at least one health condition lasting ≥12 months, rising to 45.2% for those taking part as walkers. The conditions reported match some of those for which GPs would prescribe physical activity, such as depression, anxiety, arthritis, hypertension, obesity, and being overweight. More than seven out of 10 of those surveyed who had health conditions improved their fitness, physical health, mental health, and other measures, suggesting that parkrun could also deliver some of the components of the *5 Steps to Mental Wellbeing* as advocated by the NHS.

There was a maximum of 47 questions asked and choices within some questions were randomised. This study analysed the responses to the questions given in Box 1.

**Box 1.** Survey questions analysed in this study

*Are your day-to-day activities limited because of a health condition or disability which has lasted, or is expected to last, at least 12 months? Include conditions related to old age, sensory deficits, mobility problems, developmental conditions, learning impairments, and mental health*. [Answers: No/Yes, limited a little/Yes, limited a lot/Don’t know, rather not say]. A list of 142 conditions were given plus ‘other’ where a free text response was requested; responders could select as many conditions as were applicable. *To what extent has running or walking at parkrun changed your ability to manage your health condition, disability, or illness?* [Answers: much worse/worse/no effect/better/much better]. *What motivated you to first participate at parkrun as a runner or walker*? Responders were asked to select a maximum of three answers out of a possible 20 motives plus *‘*other’ where a free text response was requested. *Thinking about the impact of parkrun on your health and wellbeing, to what extent has running or walking at parkrun changed?* [Answers: much worse/worse/no impact/better/much better]. There was a list of 15 impacts plus *‘*other’ where a free text response was requested.

Informed consent was obtained from all subjects involved in the study.

### Matching data from parkrun

Responders provided their name, unique parkrun ID number (from their parkrun barcode, allocated at parkrun registration), date of birth, and home parkrun, which allowed their survey data to be matched to parkrun databases. This provided the following information:

- date of parkrun registration;
- sex (at parkrun registration);
- Index of Multiple Deprivation (IMD) derived from postcode where IMD quartile 1 is the most deprived and IMD quartile 4 the least deprived;
- response to the following question asked at registration: *Over the last 4 weeks, how often have you done at least 30 minutes of moderate exercise (enough to raise your breathing rate)?* [Answer: less than once per week/about once per week/about twice per week/about three times per week/four or more times per week/rather not say/don’t know]. Those selecting ‘less than once per week’ were classified as ‘inactive’; and
- their mean time for completing the parkruns.

### Definition of walkers, runners/walkers, and runners

The following definitions were used in this study:

- front runners: those with mean 5 km times <20 min;
- median runners: runners in the category containing the median runner (the 22 832th runner with a time of 29 min 20 s), that is, those with mean 5 km times between 27.5 and 30 min;
- slower runners: those with mean 5 km times between 42.5 and 45 min;
- runners/walkers: those likely to have combined running and walking with mean 5 km times between 45 and 50 min; and
- walkers: those with mean 5 km times ≥50 min or a mean speed of 6 km/h(1.67 m/s).^14^

The remaining times were split into 11 categories 2.5 min apart.

### Preliminary analysis

Data were validated using Excel (version 16.46) using statistical descriptors. Data for motives and impact were coded in Excel (version 16.49) and all statistics analysed using SPSS (version 26).

### Statistical analysis

Data within each time range were reported as frequencies or medians (since the variation within each time range was non-parametric). Categorical data for each time range were compared with walkers using the χ^2^ test with effect size calculated using Cramér’s V.^15^ Continuous data were compared with walkers using the Kruskal–Wallis test with effect size defined as *r* = *z-*score/*√n* where *n* is the number of valid cases. Effect sizes were defined as small (<0.25), moderate (0.25 to 0.45), and large (>0.45). Statistical significance was set to *P*<0.001.

## RESULTS

The survey was sent to all those who had been registered with parkrun since 2004 (whether their participation had lapsed or not) and those who had never done a parkrun (around 43% of those registered), which may account for the relatively low response rate of 100 866 survey returns (around 4.4% of registrants and 7.7% of participants). The following responders were removed: 37 039 who consented to view the survey but did not answer any questions; 1786 who had registered with parkrun but had not yet participated; 1349 who did not consent; 681 who self-identified exclusively as volunteers; and 12 who provided invalid or malicious responses. This left 59 999 responses, of which approximately 75% were matched to parkrun data, resulting in 45 662 participants with matched mean 5 km times from the parkrun database.

### Demographics

**Figure 1.**
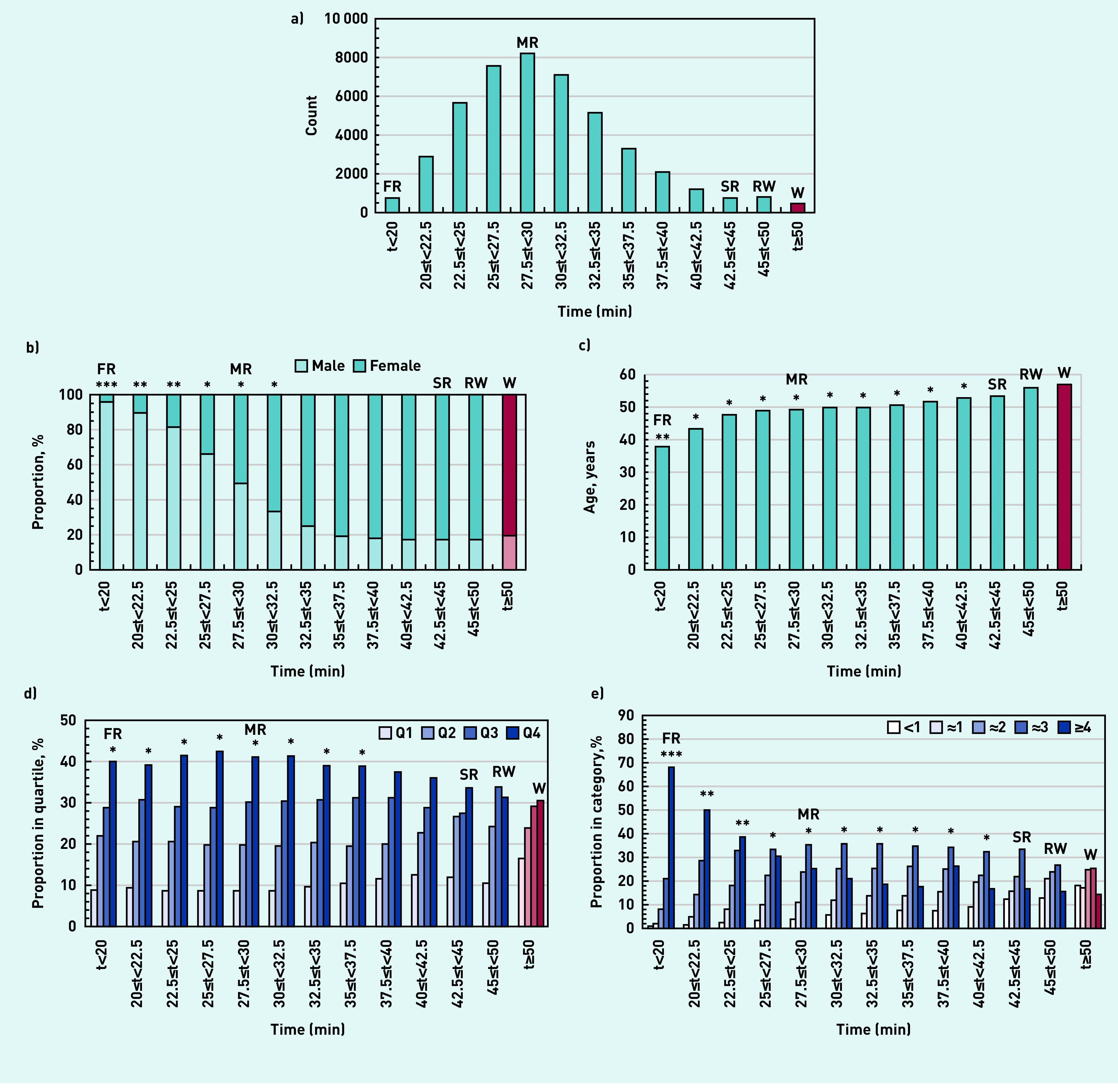
*Characteristics of survey participants ranked by average running time: a) count; b) proportion male and female; c) age; d) Index of Multiple Deprivation quartile (Q1 is most deprived); e) activity level at registration in bouts of 30 min or more in previous 4 weeks. Comparison with walkers at* P <0.001 with effect sizes: *small, **moderate, ***large. Red and pink bars represent walkers. FR = front runners. MR = median runners. RW = runners/walkers. SR = slower runners. W = walkers.

Figure 1 shows the demographics of participants ranked by average running time (see Supplementary Table S1 for details). In comparison with the full parkrun population, the sample had a similar proportion of females (51.7% for the sample versus 51.3% for the population), a similar ethnic and employment background, and was older (48.0 years for the sample versus 40.5 years for the population).^13^ The latter was primarily because the survey was restricted to those aged ≥16 years.

Responders were normally distributed about a median of 27.5 to 30 min but with a tail of slower runners, runners/walkers, and walkers (Figure 1a). Of the sample, 51.5% were female, ranging from 4.2% for front runners to 80.3% for walkers (Figure 1b). The median age increased from 37.8 years for front runners to 56.9 years for walkers (Figure 1c). There were fewest participants from IMD Q1 (most deprived areas) and most from IMD Q4 (least deprived areas), with walkers more likely to be from deprived communities (Figure 1d). Around one-third of slower runners, runners/walkers, or walkers were inactive or did about one bout of activity per week at registration (Figure 1e).

Those faster than median runners showed significant demographic differences from walkers with large effect sizes. Slower runners and runners/walkers were statistically similar to walkers and were more likely to be female, older, from deprived communities, and less active at registration.

### Health conditions

**Figure 2.**
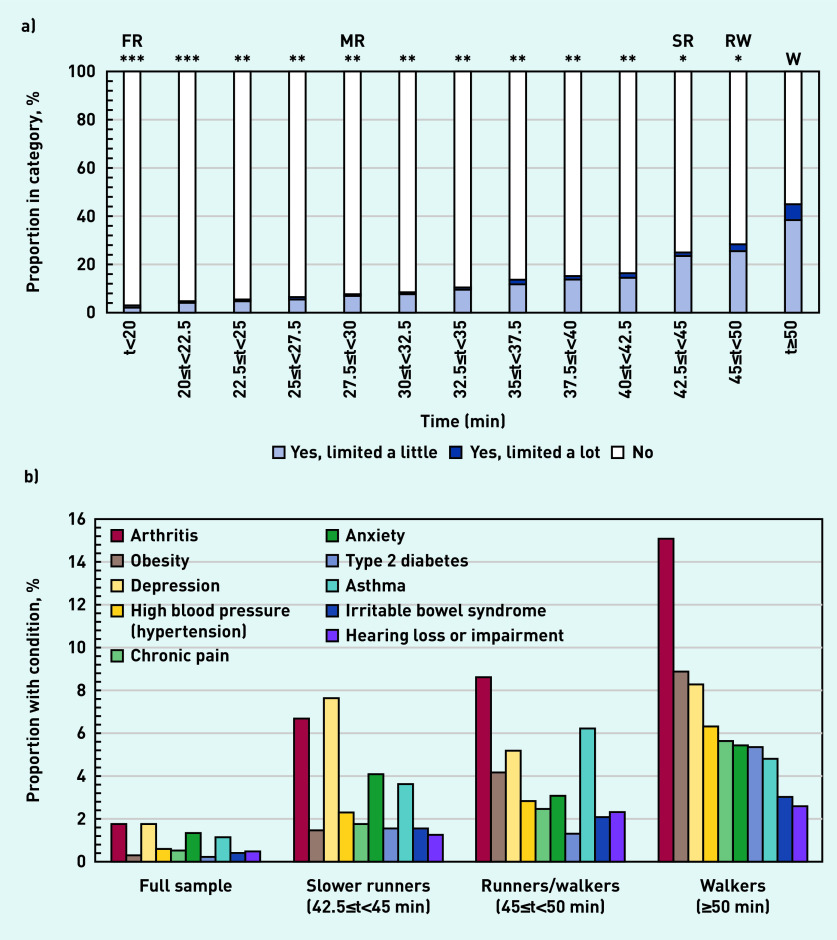
Characteristics of survey participants ranked by average running time: a) proportion limited by a health condition for ≥12 months; and b) proportion with each health condition (only top 10 conditions shown). Note: participants could have more than one health condition. Comparison with walkers using χ^2^ test at P<0.001 with effect sizes: *small, **moderate, ***large. FR = front runners. MR = median runners. RW = runners/walkers. SR = slower runners. W = walkers.

Figure 2 gives the characteristics of survey participants with health conditions ranked by average running time. Figure 2a shows that the proportion limited by at least one health condition lasting ≥12 months rose from 3% for front runners to 25% for slower runners, 28% for runners/walkers, and 45% for walkers. The overall proportion for the full sample was 9.3% (see Supplementary Table S1 for details). Slower runners, runners/walkers, and walkers had a median of two health conditions compared with a median of one health condition for the full sample. Slower runners, runners/walkers, and walkers collectively represented 4.3% of the sample and reported 19.8% of health conditions. The most reported conditions are shown in Figure 2b (see Supplementary Table S1 for details). For the full sample, the top five conditions were depression, arthritis, anxiety, asthma, and hypertension; slower runners, runners/walkers, and walkers also reported fibromyalgia, obesity, and chronic pain.

### Motives for first participating and impact following participation

Supplementary Figure S1 illustrates responders’ motives for first participating in parkrun paired, where possible, with impact measures (see Supplementary Table S2 for details). The graphs are ranked in order of most to least selected motive for the full sample.

The three most selected motives were ‘to contribute to my fitness’ (57.0%), ‘to improve my physical health’ (37.2%), and ‘to gain a sense of personal achievement’ (27.2%); these had large proportions of people reporting improvements of 90.1%, 85.4%, and 91.4%, respectively.

Fewer slower runners, runner/walkers, and walkers selected ‘to contribute to my fitness’, while more selected ‘to improve my physical health’.

‘To manage my weight’ was selected by 19.6% of the sample and was more likely to be selected by slower runners (33.8%), runners/walkers (33.0%), and walkers (32.7%), with improvement for approximately 55% of runners with times slower than the median.

‘To improve or manage my health condition, disability, or illness’ was selected by 17.4% of those with a health condition and was more likely to be selected by walkers (31.5%). A total of 66.8% of all responders reported improvements to ‘your ability to manage your health condition, disability, or illness’, with no statistical differences between walkers and other participants.

Few selected as a motive ‘to improve my mental health’ (12.7%), ‘to feel part of a community’ (11.3%), or ‘to improve my happiness’ (6.5%). However, large proportions of responders reported improvements in these areas: 69.5%, 71.1%, and 79.6%, respectively. There were few statistical differences between walkers and other responders.

Few responders selected ‘to spend time outdoors’ (10.0%) or ‘to be active in a safe environment’ (3.9%), although the former was statistically more likely to be selected by walkers and the latter by runners slower than the median. ‘The amount of time you spend outdoors’ was improved for 74.8%, while ‘your ability to be active in a safe environment’ was improved for 60.0% of participants. There were higher values for walkers at 81.8% and 71.3%, respectively.

More than 20% of slower runners, runners/walkers, and walkers were more likely to select ‘my friends, family, or colleagues encouraged me to’ and, while more walkers selected ‘a health professional advised me to’, this was only 1.8% compared with 0.3% for the full sample. (It should be noted that the survey was carried out as parkrun practice was being set up.) Finally, 51.9% of the full sample improved ‘your overall lifestyle choices (for example, diet and smoking)’, with little difference between walkers and other responders.

## DISCUSSION

### Summary

In a survey of 45 662 parkrunners, slower runners tended to be older, and were more likely to be female, from a deprived community, and inactive at registration. More than 9% of the full sample were found to have at least one long-term health condition lasting ≥12 months: this rose to 45% for walkers. While slower runners, walkers/runners, and walkers represented 4.3% of participants, they reported 19.8% of health conditions: these conditions included arthritis, anxiety, asthma, depression, chronic pain, fibromyalgia, hypertension, and obesity.

Slower runners, runners/walkers, and walkers were less likely to be motivated by fitness or competition than other parkrunners and more likely to be motivated by physical health, weight management, the management of their health condition(s), to spend time outdoors, and to be active in a safe environment. Despite these differences, perceived improvements to wellbeing were broadly similar, regardless of the responder’s finishing time.

### Strengths and limitations

The analysis is drawn from a large survey, allowing statistically significant differences to be found between categories of runner/walker. Any survey is biased by the responders who answer it: in this case, responders might be considered ‘keen’ parkrunners with fewer health conditions than the general population, and they may be more likely to report improvements. Sex is a confounding factor in the analysis so that, for runners slower than the median, motives and impact may reflect the views of females rather than males. Other confounding factors are age, IMD, activity level at registration, and parkrun participation.

### Comparison with existing literature

As with previous studies,^16^^–^^19^ the current study has shown that walking can confer similar health benefits to running. Fleming *et al* ^11^ found that parkrun practices suggested to patients that participation could be through jogging or walking. This study shows that those with health conditions may already be participating in this way. The health benefits of parkrun have previously been studied,^20^^–^^24^ and this work confirms a 2015 study,^20^ which found that large proportions of participants improved wellbeing measures, with non-runners more likely to improve than runners. Another study involving the parkrun survey^24^ found that volunteering could also improve wellbeing and suggested that parkrun could deliver some of the components of *5 Steps to Mental Wellbeing* promoted by the NHS.^25^ The results of the current study show that this is also true of running or walking.

### Implications for practice

This article provides the rationale to general practice staff for signposting to parkrun, by outlining the broad range of people that take part as walkers and runners, what motivates them, and the impact to them of participation. Faster runners are very different from the slowest, although they still perceive some of the same wellbeing benefits. Forty-five per cent of walkers reported long-term health conditions, some of which are those for which GPs say they would prescribe physical activity: depression, anxiety, hypertension, obesity, and being overweight.^1^^,^^5^^,^^6^^,^^12^ When discussing potential benefits with patients, messages may include obvious impacts such as improvements to fitness and physical health. However, while few responders chose mental health, happiness, or feeling part of a community as a motive to join parkrun, seven to eight out of 10 responders reported improvements in these areas, with little difference between walkers and other runners. These areas may be equally important to those new to activity as well as to those who are already active but who might benefit from improved mental health. The information provided in this study should be combined with other research investigating the barriers to participation in parkrun.^26^ A toolkit could be provided via parkrun practice^10^ that incorporates the latest knowledge about delivering brief physical activity interventions in primary care^27^ to help address the key issues of clinicians’ knowledge and confidence.

## Data

The datasets supporting the conclusions of this article are stored in the Sheffield Hallam University Research Database (SHURDA: DOI: http://doi.org/10.17032/shu-180037). The full anonymised dataset is also accessible to researchers for research purposes through the parkrun research board, as originally outlined in the participant information sheet.
